# Coevolution within and between Regulatory Loci Can Preserve Promoter Function Despite Evolutionary Rate Acceleration

**DOI:** 10.1371/journal.pgen.1002961

**Published:** 2012-09-20

**Authors:** Antoine Barrière, Kacy L. Gordon, Ilya Ruvinsky

**Affiliations:** 1 Department of Ecology and Evolution and Institute for Genomics and Systems Biology, The University of Chicago, Chicago, Illinois, United States of America; 2 Department of Organismal Biology and Anatomy, The University of Chicago, Chicago, Illinois, United States of America; Fred Hutchinson Cancer Research Center, United States of America

## Abstract

Phenotypes that appear to be conserved could be maintained not only by strong purifying selection on the underlying genetic systems, but also by stabilizing selection acting via compensatory mutations with balanced effects. Such coevolution has been invoked to explain experimental results, but has rarely been the focus of study. Conserved expression driven by the *unc-47* promoters of *Caenorhabditis elegans* and *C. briggsae* persists despite divergence *within* a *cis*-regulatory element and *between* this element and the *trans*-regulatory environment. Compensatory changes in *cis* and *trans* are revealed when these promoters are used to drive expression in the other species. Functional changes in the *C. briggsae* promoter, which has experienced accelerated sequence evolution, did not lead to alteration of gene expression in its endogenous environment. Coevolution among promoter elements suggests that complex epistatic interactions within *cis*-regulatory elements may facilitate their divergence. Our results offer a detailed picture of regulatory evolution in which subtle, lineage-specific, and compensatory modifications of interacting *cis* and *trans* regulators together maintain conserved gene expression patterns.

## Author Summary

Some phenotypes, including gene expression patterns, are conserved between distantly related species. However, the molecular bases of those phenotypes are not necessarily conserved. Instead, regulatory DNA sequences and the proteins with which they interact can change over time with balanced effects, preserving expression patterns and concealing regulatory divergence. Coevolution between interacting molecules makes gene regulation highly species-specific, and it can be detected when the *cis*-regulatory DNA of one species is used to drive expression in another species. In this way, we identified regions of the *C. elegans* and *C. briggsae unc-47* promoters that have coevolved with the lineage-specific *trans*-regulatory environments of these organisms. The *C. briggsae* promoter experienced accelerated sequence change relative to related species. All of this evolution occurred without changing the expression pattern driven by the promoter in its endogenous environment.

## Introduction

Conserved patterns of gene expression, especially among closely related species, immediately suggest conservation of the regulatory mechanisms that bring them about. However, considerable sequence divergence has been documented in orthologous regulatory elements [1], [2] and turnover of experimentally validated transcription factor binding sites is known to occur [3], [4] and to be selected on [5]. Such *cis*-regulatory changes occur on their own, or coevolve with transcription factors that regulate them [6]–[8], and with chromatin modifiers [9]. Indeed, entire regulatory networks that are crucial for organismal survival nonetheless vary within [10], [11] and between species [12]–[14].

Since evolutionary biologists are interested in species divergence, most studies of regulatory evolution focus on gene expression differences between two species or strains [15]–[17], which is essential for understanding the molecular processes by which evolution occurs. However, the necessary counterpart to studies of differentially expressed genes are studies that address how, despite the inexorable evolution of genome sequence, some genes retain conserved expression. This second category comprises a substantial fraction of genes; for instance, only about a quarter of genes show expression differences between strains of yeast [18] or Drosophila [19], and over a third of orthologs show conserved expression even among distantly related vertebrates [20]. We want to understand how expression conservation is achieved.

One possibility is that purifying selection preserves functional elements, which are nestled within functionless and divergent sequences [21]. Another possibility is that regulatory functions can be carried out by degenerate sequences that can sustain substantial substitution without altering their conserved function [22]. Yet another possibility is that coevolutionary changes among the multiple regulators of a single gene compensate for one another to maintain a conserved output [23]–[25]. This scenario is only detectable in a comparative context—expression patterns must be conserved while the specific interactions among regulatory molecules diverge in one organism relative to another.

One way to document this phenomenon is to perform functional comparisons of orthologous *cis*-regulatory elements in a common *trans* background. This can be done in several ways. Diverged regulatory elements can be introduced into a hybrid *trans* environment on the genome-wide scale by interspecific crosses, after which allele-specific expression can be measured by microarray [26], [27], sequencing of individual genes [28], or high-throughput sequencing [29], [30]. Such methods have the advantage of assaying multiple loci at once and detecting genome-wide regulatory divergence. These approaches are useful for identifying the molecular underpinnings of hybrid incompatibility [31]. In some cases, QTL studies can be used to uncover genomic sequences associated with gene expression differences [32], [33]. Follow-up experiments can then determine the molecular effects of associated mutations on expression [34], [35].

Transgenic methods provide an approach to studying the loci of regulatory evolution in a controlled, experimentally manipulable way. Single regulatory elements from the genome of one species can be introduced into a host of a pure-species, rather than hybrid, background. While this method can only be used to dissect one regulatory element at a time, it has the advantages of being tractable in non-hybridizing species, isolating particular molecular interactions between *cis* and *trans*, and allowing experimental manipulations of regulatory sequence and genetic background to isolate mechanisms of action [36]. When a pair of organisms are both amenable to transgenesis, a highly controlled experiment of reciprocal *cis*-regulatory element swaps can be performed [37]–[43].

This necessary quality is found in *Caenorhabditis elegans* and *C. briggsae*, two nematodes with considerable sequence divergence [44] and morphological conservation. Here, we studied the *cis*-regulatory element of the *unc-47* gene, which has a simple and quantifiable expression pattern [22], [45]. We examined the functions of regulatory sequences from one species in the other to discern whether lineage-specific *cis-trans* and *cis*-*cis* interactions have evolved.

## Results

### Divergent *cis*- and *trans*-regulatory information underlies a conserved expression pattern

**Figure 1:**
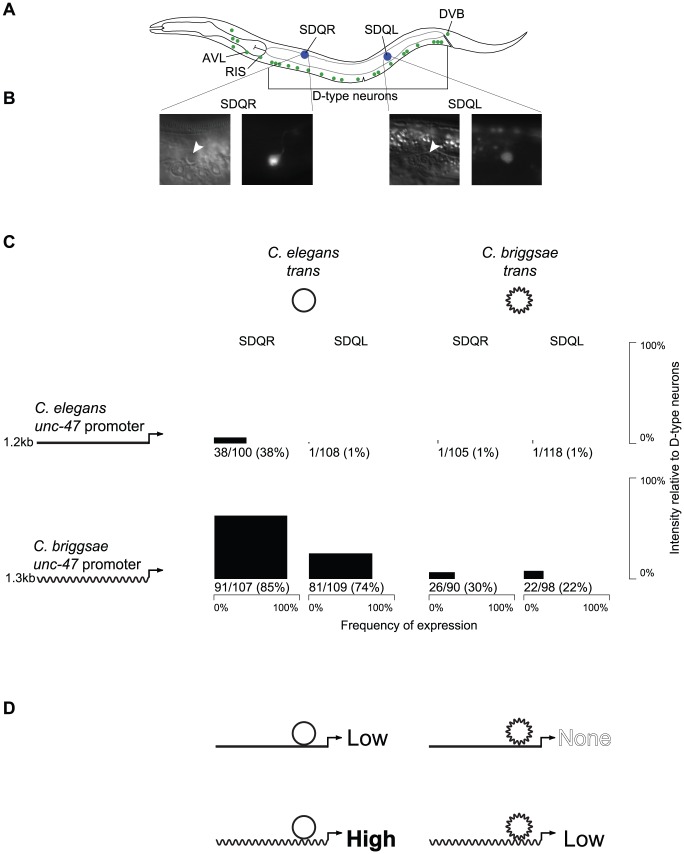
The expression pattern of *unc-47* is conserved despite divergent regulation. (A) Both *C. elegans* and *C. briggsae* promoters fused upstream of GFP in their endogenous *trans*-regulatory environments drive expression in all 26 GABAergic neurons (green). However, the *C. briggsae* promoter placed in the *C. elegans trans* environment additionally drives expression in SDQR and SDQL (blue). (B) Those cells were identified as SDQR/L based on their position and their characteristic projections. (C) For each combination of promoter and *trans*-regulatory environment, expression in SDQR and SDQL is presented. *C. elegans* is represented by straight lines, *C. briggsae* by wavy lines. Frequency of expression is represented by the width, and intensity of expression relative to D-type neurons by the height of black boxes. Number of individuals expressing and total number of individuals scored is indicated underneath. Measurements for independent strains are given in Figure S1. Differences in frequency of expression in SDQR are significant for all comparisons: *C. elegans* and *C. briggsae* promoters in *C. elegans trans* environment (p = 8.1×10^−12^), *C. elegans* and *C. briggsae* promoters in *C. briggsae trans* environment (p = 1.5×10^−8^), *C. elegans* promoter in *C. elegans* and *C. briggsae trans* environments (p = 4.8×10^−11^), *C. briggsae* promoter in *C. elegans* and *C. briggsae trans* environments (p = 2.4×10^−14^). (D) Interpretation of the results presented in panel C. Both *C. elegans* and *C. briggsae* promoters in their endogenous *trans* environments drive low levels of expression in SDQR and SDQL, while disruption of the endogenous interactions either drives high levels of expression (*C. briggsae* promoter in *C. elegans*) or abolishes expression (*C. elegans* promoter in *C. briggsae*).

The nervous system of *C. elegans* contains 26 GABAergic neurons ([46], Figure 1A), which are conserved with even distantly related nematodes [47]. The expression patterns of genes involved in defining the identity of GABAergic neurons, such as the vesicular GABA transporter *unc-47* [48], are expected to be conserved as well. This expectation can readily be tested in *C. elegans* and *C. briggsae*, two closely related species that have nearly identical embryonic cell lineages [49], which allows for homology of individual cells to be unambiguously assigned. Indeed, *cis*-regulatory elements of *C. elegans* and *C. briggsae unc-47* genes direct almost identical expression patterns in their respective *trans*-regulatory environments [45].

To test whether expression conservation results from conserved regulatory mechanisms, or from lineage-specific compensatory evolution, we performed reciprocal transgenic experiments using GFP reporters fused to regulatory elements of *C. elegans* and *C. briggsae unc-47* genes. Although animals of all four possible *cis* by *trans* combinations expressed GFP in all GABAergic neurons, there were notable differences between them as well. In addition to expression in all 26 GABAergic neurons, GFP was also sometimes expressed in two other neurons, SDQR and SDQL (Figure 1A). Identity of these cells was definitively established based on their positions and the appearance of their projections (Figure 1B).

Both *C. elegans* and *C. briggsae* promoters fused to GFP and introduced as extrachromosomal arrays into their endogenous environments appear to drive very weak expression in SDQR/L (about 20-fold less intense than in GABAergic neurons) in around one-third of individuals (Figure 1C). While it is not thought that *unc-47* is endogenously expressed in these non-GABAergic neurons, both promoters drive weak expression in SDQR/L in a minority of animals in a fashion that is consistent across independent strains (Figure S1).

However, when the *cis* element of one species is expressed in a transgenic host of the other species, expression in SDQR/L is very different. The *C. elegans unc-47* promoter is almost never observed to drive expression in transgenic *C. briggsae* animals. On the other hand, the *C. briggsae unc-47* promoter drives strong, consistent expression of GFP in most *C. elegans* animals carrying the transgene (Figure 1C and Figure S1). We infer that the strong expression in these cells results from mismatched *cis*-regulatory information of the *C. briggsae* promoter and *trans*-regulatory information in *C. elegans*.

To confirm that the expression differences we observed are due to different activities of the *cis*-regulatory elements in the different host backgrounds and are not artifacts of the extrachromosomal array method that we used, we performed additional experiments. First, we made sure that the patterns of expression we report are consistent across multiple strains bearing independently generated extrachromosomal arrays (Figure S1). Second, we integrated *unc-47::GFP* promoter fusions into the genomes of *C. elegans* and *C. briggsae*, and verified their expression consistency across multiple independent strains (Figure S2). Third, we utilized MosSCI technology [50], which is available only in *C. elegans*, to generate single-copy integrants of the *C. elegans* and *C. briggsae unc-47::GFP* transgenes into the same genomic locus (Figure S3). Both the direction and approximate magnitude of the expression differences between the four possible combinations of *cis* and *trans* are consistent among all of these transgenic methods, and between independent lines generated using the same method (Figure 1C; Figures S1, S2, and S3). These data overwhelmingly support the hypothesis that misexpression in SDQR/L is the result of interactions between divergent *C. elegans* and *C. briggsae cis* and *trans* regulators, and is not an experimental artifact.

In many cases, the effect of combining *cis* and *trans* elements from different species leads to misregulation of gene expression [36]. When such divergence occurs while preserving major characteristics of a phenotype (be it a morphological trait [51] or a gene expression pattern [52]), it is called Developmental Systems Drift [24]. Far from being meaningless experimental artifacts, these cases of misexpression reveal evolutionary divergence in regulatory components that would otherwise go undetected due to the conservation of their phenotypic output. This type of divergence [53], which leads to negative epistatic interactions, is evolutionarily significant, as it could create Dobzhansky-Muller Incompatibilities ([31], the genetic interactions that go awry in hybrids and keep species separate from one another [54]. In fact, the pattern that we observe in Figure 1C is reminiscent of the pattern that appears in cases of transgressive segregation [8], [55]–[57], in which hybrid phenotypes exceed parental values for a quantitative trait as a result of interactions between divergent elements in the parental genomes. As has been noted [58], there are compelling observations from both flies [8] and yeast [27] that the *cis-trans* coevolution often involves changes with the opposite effect on gene expression, perhaps as a result of balancing selection on gene expression favoring the fixation of compensatory mutations.

We therefore propose an explanation (Figure 1D) for our observations that is informed by the rich literature on misexpression of heterologous transgenes and hybrid dysregulation. We hypothesize that the conserved gene expression pattern of *unc-47* in *C. elegans* and *C. briggsae* is the result of lineage-specific coevolution in which *C. elegans* balances the effects of a relatively weaker *cis*-regulatory element and a stronger *trans*-regulatory environment in SDQR/L to produce the conserved output, while *C. briggsae* balances a stronger *cis*-regulatory element with a weaker *trans*-regulatory environment in these cells. Therefore, in either host (compare down columns of Figure 1C), the *C. briggsae cis* element always drives stronger expression in SDQR/L than the *C. elegans* ortholog (Figure 1C; chi-squared test for difference in frequency of expression in SDQR between the two promoters in *C. elegans* p = 8.1×10^−12^; in *C. briggsae* p = 1.5×10^−8^). The *trans* environment of a *C. elegans* host animal always drives stronger expression in these cells than the *trans* environment of a *C. briggsae* host (compare across rows of Figure 1C; chi-squared test for difference in frequency of expression in SDQR between the two *trans* environments of the *C. elegans* promoter p = 4.8×10^−11^; of the *C. briggsae* promoter p = 2.4×10^−14^). Only when the *cis* and *trans* regulators of *unc-47* from different species are combined experimentally can their different functions be observed.

### A conserved regulatory motif is necessary for expression in SDQR/L and DVB

**Figure 2:**
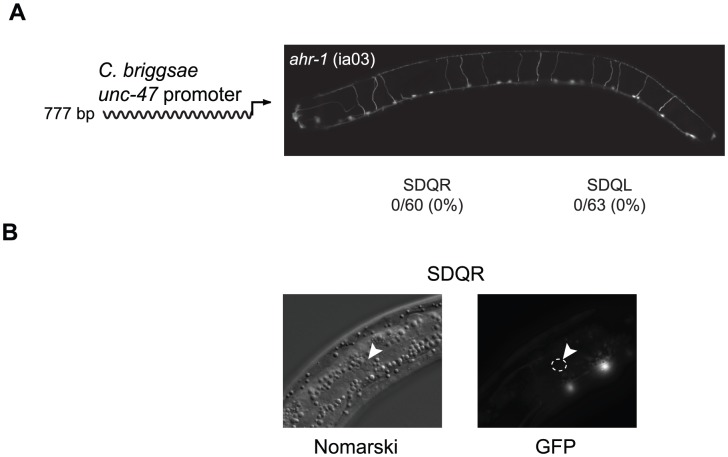
Expression of the *C. briggsae unc-47::GFP* transgene in SDQR/L requires AHR-1. (A) In an *ahr-1* loss-of-function mutant (ia03), GFP is not expressed in SDQR/L. Number of individuals expressing and total number of individuals scored is indicated. Expression of the 777 bp promoter in WT background is shown in Figure 3A. (B) SDQR/L are indeed present in the *ahr-1* (ia03) mutant as can be seen by Nomarski, but are not expressing GFP. SDQR is shown.

To identify the coevolved *cis* and *trans* regulators, we searched for transcription factors that are expressed in SDQR/L. The gene *ahr-1*, which encodes a bHLH transcription factor [59], is expressed in a number of neurons including SDQR/L [60]. It is known to regulate the fate of some GABAergic [61] as well as other [62] neurons. In *C. elegans ahr-1* (ia03) mutants, expression of a *C. briggsae unc-47* promoter in SDQR/L was completely abolished (Figure 2A), even though the cells were still present (Figure 2B and [62]). This experiment demonstrates that *ahr-1* is necessary for expression in the cells in which ectopic expression is observed. To test whether *ahr-1* is also the site of *trans*-regulatory divergence, we conducted several experiments. Our results find expression differences (via qRT-PCR and transgenic expression assays, data not shown) and coding sequence differences between the species that imply, but do not prove, that the function of *ahr-1* has diverged between *C. elegans* and *C. briggsae* and could affect *unc-47* regulation.

However, *trans*-regulatory differences are more difficult to identify than *cis*-regulatory differences. A causal *cis*-regulatory change must be located within the DNA that has divergent function (in this case, ∼1.3 kb of DNA upstream of *unc-47*). On the other hand, evidence for change in *trans* potentially implicates the entire genome. The causal nucleotide changes between *C. elegans* and *C. briggsae* could potentially reside in coding or regulatory sequence of *ahr-1*, an upstream regulator of *ahr-1*, a binding partner or antagonist of *ahr-1*, or in multiple interacting loci. However, we can use the *ahr-1* clue to dissect the mechanism of *cis*-regulatory divergence.

**Figure 3:**
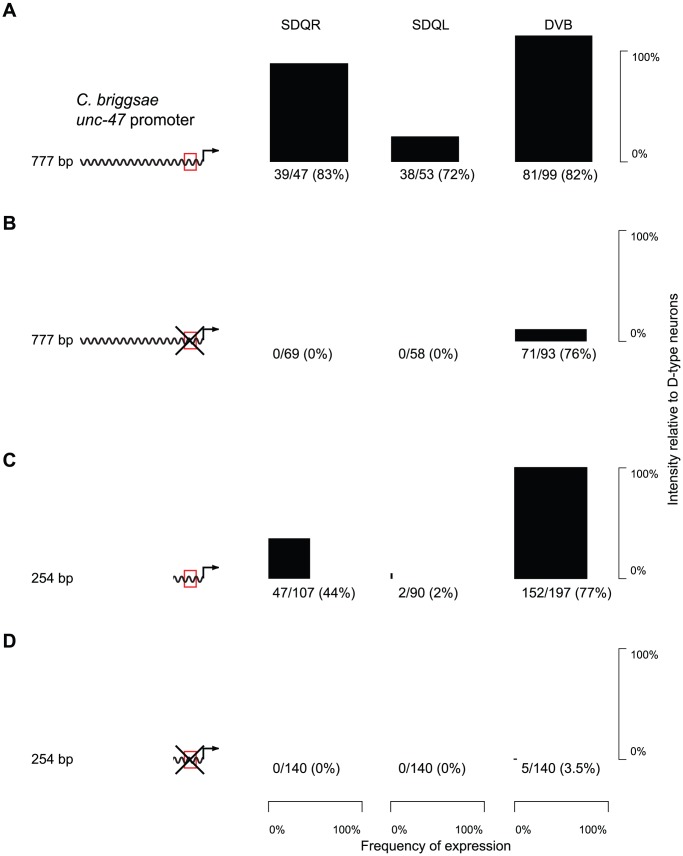
Expression in SDQR/L is mediated by a conserved motif, which also controls expression in DVB. (A–D) GFP expression in SDQR, SDQL and DVB driven by *cis* elements, with an intact (red box; A, C) or mutated (crossed red box; B, D) AHR-1 core consensus motif. Frequency of expression is represented by the width, and intensity of expression relative to D-type neurons by the height of black boxes. Number of individuals expressing and total number of individuals scored is indicated underneath. The difference between distributions of DVB intensity in panels A and B is highly significant (p<2.2×10^−16^). Counts for multiple independent strains are given in Table S1.

The core binding sequence of AHR-1 has been experimentally defined as CACGC [63] or CACGCA [59]. There is a single occurrence of such a motif in a conserved block of sequence within the proximal promoter of *unc-47* (Figure S4). Whereas a *C. briggsae* promoter with the AHR-1 core consensus site intact drove strong and consistent expression in *C. elegans* SDQR/L (Figure 3A and Table S1), a mutation of this putative binding site completely abrogated expression in these two, but not other neurons (Figure 3B and Table S1). Based on this evidence we concluded that the SDQR/L expression of the *C. briggsae unc-47* promoter is regulated through this site. But does it have a regulatory function with respect to expression in GABAergic neurons?

Worms carrying the mutated promoter showed significantly less intense expression in DVB (compare Figure 3A and 3B, Kolmogorov-Smirnov test p<2.2×10^−16^). We interpreted this to mean that endogenous expression of *unc-47* in DVB is controlled via this motif as well as one or more additional sequences. The distal promoters of *unc-47* are highly divergent in their sequences and contribute to the robustness of expression in DVB [22]. We excluded them as candidates for the sequence that directs expression in SDQR/L and DVB by examining expression of the proximal promoter alone. The proximal promoter with the intact motif drove expression in SDQR/L and DVB similarly to the full-length promoter, albeit less intensely (Figure 3C and Table S1). In contrast, the mutated proximal promoter showed essentially no SDQR/L or DVB expression (Figure 3D and Table S1). The proximal promoter must therefore be the site of *cis*-regulatory change that maintains expression in DVB via the conserved core consensus motif and has pleiotropic effects on expression in SDQR/L when the promoters are swapped between species.

### Extensive epistasis within the proximal promoter of *unc-47*

**Figure 4:**
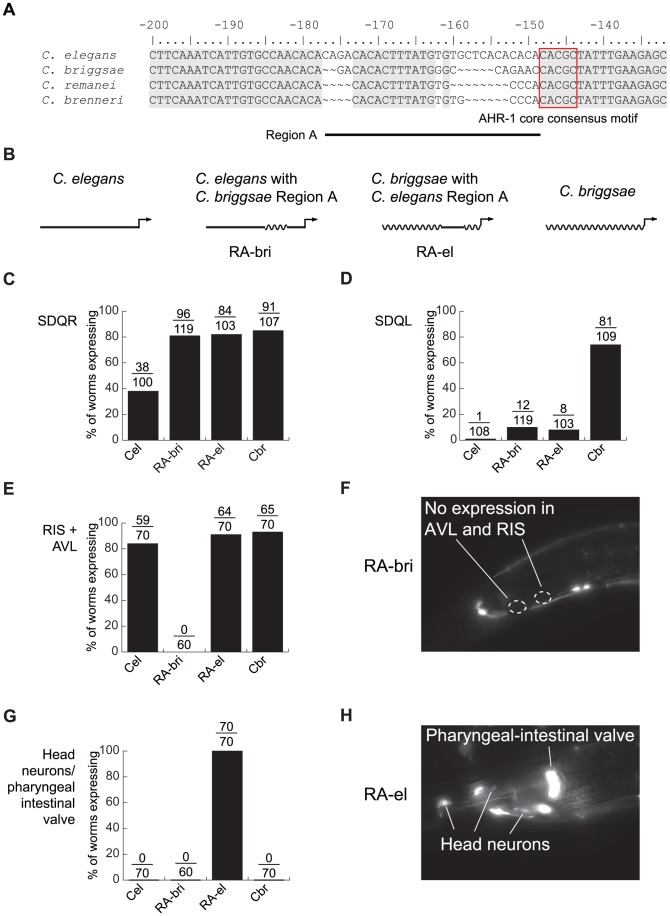
Lineage-specific coevolution within *unc-47* promoters. (A) An alignment of the sequences flanking the conserved AHR-1 core consensus motif (boxed in red). Regions conserved between all four species are shaded in gray. (B) Schematic representation of *C. elegans* and *C. briggsae* promoters and chimeric transgenes reciprocally exchanging Region A. (C–H) Expression driven by the four transgenes. Percentage of worms expressing GFP in (C) SDQR, (D) SDQL, (E) AVL and RIS, (G) head neurons and pharyngeal-intestinal valve. Representative pictures of (F) an individual carrying the RA-bri transgene, showing absence of expression in AVL and RIS, and (H) an individual carrying the RA-el transgene, showing strong expression in non-GABAergic head neurons and the pharyngeal-intestinal valve.

The AHR-1 core consensus motif is conserved between *C. elegans* and *C. briggsae*, so it is clearly not the site of *cis*-regulatory divergence. Because nucleotides flanking transcription factor binding sites can substantially contribute to affinity and specificity of binding [64], [65], we next concentrated on sequences in the vicinity of this motif. Differences between *C. elegans* and *C. briggsae* in this region are particularly good candidates to mediate functional divergence. We designated as “Region A” approximately 30 bp containing two divergent sequences interrupted by 12 conserved nucleotides (Figure 4A). Because regulatory sequence divergence can be buffered [66], we tested the effects of Region A divergence experimentally.

Chimeric *cis*-regulatory elements that combine segments from orthologous promoters are powerful tools for detecting lineage-specific divergence that is difficult to reveal by other approaches [42], [67]. To test whether sequences within Region A have functionally diverged between *C. elegans* and *C. briggsae*, we generated reciprocal chimeric transgenes containing either most of the *C. elegans* promoter with Region A of *C. briggsae* (RA-bri) or vice versa (RA-el, Figure 4B; exact sequences shown in Figure S5). Because Region A is flanked by extended blocks of conservation, we can be sure that the swapped DNA in these chimeric promoters is indeed orthologous. We compared expression patterns of chimeric promoters to those of the intact full-length promoters from *C. elegans* and *C. briggsae*.

In SDQR, expression from both chimeric promoters was similar to that of the *C. briggsae* promoter (Figure 4C), suggesting that the *C. briggsae* promoter contains at least two elements that control expression in this cell—one in Region A and one outside of it. In SDQL, both chimeric transgenes drove expression similar to that of the *C. elegans*, not the *C. briggsae*, promoter (Figure 4D). This suggests that the *C. briggsae*-like expression is a consequence of a synergistic epistasis between two elements—one inside Region A, another outside of it. All functional differences between Regions A of *C. elegans* and *C. briggsae* reside in a shorter, approximately 15 bp region immediately upstream of the conserved motif (Figure S5).

Our results also suggest that the endogenous function of Region A is to control aspects of the conserved GABAergic expression of *unc-47*. Because both chimeric promoters directed expression different from this conserved pattern, we inferred that the promoters experienced lineage-specific *cis-cis* coevolution. Specifically, both *C. elegans* and *C. briggsae* promoters drove strong and consistent expression in RIS and AVL, two GABAergic neurons located near the posterior bulb of the pharynx (Figure 1A). RA-bri showed no detectable expression in RIS and very little in AVL (Figure 4E and 4F, and Figure S5), suggesting that a lineage-specific interaction between an element in Region A and another one outside of it is disrupted in this chimera. Disruption of a similar interaction in RA-el caused aberrant but strong expression in a group of 8–10 non-GABAergic head neurons and in the pharyngeal-intestinal valve, a non-neuronal cell type (Figure 4G and 4H, and Figure S5). No intact promoters drove expression in these cells.

Epistasis between *cis*-regulatory sites, such as we found in the *unc-47* promoter, is not unprecedented. Intra-molecular epistatic interactions and evidence of coevolution have been observed in *cis*-regulatory elements [68] and proteins [69], [70]; they may have arisen via compensatory, pseudocompensatory, or other processes [71], [72]. Next, we sought to identify when these epistatic interactions evolved.

### Functional divergence of *unc-47* regulation occurred along the *C. briggsae* lineage

**Figure 5:**
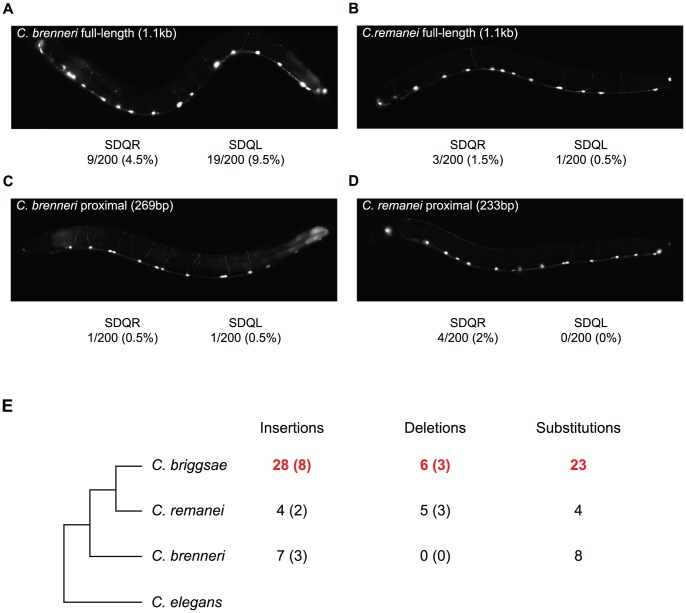
The *C. briggsae unc-47* promoter has experienced lineage-specific sequence evolution and functional divergence. (A–D) Fluorescence images of *C. elegans* individuals carrying (A, C) *C. brenneri*, (B, D) *C. remanei*, (A, B) full-length, and (C, D) proximal promoter-GFP fusion transgenes. The percentage of individuals expressing GFP is given. In all cases when expression was visible it was weak compared to the GABAergic neurons. (E) The number of nucleotides inferred to be lineage-specific changes in the proximal promoter of *unc-47*. The number of indel events are shown in parentheses. Sites conserved between two species but divergent in the third were counted as lineage-specific. Details are shown in Figure S6.

To determine when in the evolutionary history of these nematodes the *C. briggsae*-like function of the proximal promoter arose, we compared the function of the *unc-47* promoter from two additional species, *C. brenneri* and *C. remanei*. These *unc-47* promoters do not drive much expression in SDQR/L in transgenic *C. elegans* (Figure 5A–5D), meaning that the functional evolution we observed occurred specifically in the *C. briggsae* lineage. Is this functional divergence reflected in the evolution of the promoter's sequence?

We compared the *unc-47* proximal promoters in a phylogenetic context that includes the two additional species (Figure 5E) in order to assign changes in the promoter sequence to a particular lineage. We observed an excess of insertions and substitutions on the branch leading to *C. briggsae* (Figure 5E and Figure S6). This pattern is particularly striking in a region of ∼160 bp in which one fifth of *C. briggsae* positions are derived (Figure S6), while *C. brenneri* and *C. remanei* do not show any lineage-specific differences. Accelerated rate of sequence change is restricted to the promoter; the rates of divergence in the protein-coding portion of the gene are the same for all species. Compared to the 487aa *C. elegans* protein sequence, *C. briggsae* UNC-47 differs at 51 positions, *C. remanei* at 47, and *C. brenneri* at 50. Rates of nonsynonymous substitutions were also similar when *C. elegans* sequence was compared to the other three orthologs (*K_a_* = 0.07, 0.07, 0.06, respectively). Whereas *cis* element evolutionary rate accelerations associated with phenotypic change are well-documented [68], [73], [74], in this case no overt phenotypic divergence seems to be linked to the acceleration of regulatory sequence evolution.

## Discussion

Comparative functional and sequence data yielded a picture of evolution of *unc-47* regulation in Caenorhabditis nematodes. Although the endogenous patterns of expression remain unchanged, the mechanisms responsible for maintaining them seem to have diverged in the *C. briggsae* lineage, possibly as a consequence of substantial divergence in the regulatory sequence. Because the *C. briggsae* promoter does not drive strong expression in SDQR/L in its endogenous *trans* environment despite its dramatic sequence evolution, a compensatory *trans* change must be inferred.

A simple model suggests the types of coevolutionary changes that were involved (Figure 1D). In their endogenous *trans*-regulatory environments, both *C. elegans* and *C. briggsae* promoters of *unc-47* drive similar weak expression in SDQR and SDQL. This similarity cannot be due to conservation of the underlying regulatory system, given the difference in expression patterns of reciprocally swapped promoters. The *C. elegans* promoter directed virtually no expression in SDQR/L of *C. briggsae*, while the *C. briggsae* promoter was strongly expressed in these cells when placed in *C. elegans*. At least two lineage-specific changes must have occurred since the divergence of *C. elegans* and *C. briggsae*: one in the *unc-47* promoter and another, possibly in a transcription factor that controls its expression. Similar cases have been documented in yeast [75] and animals [68], [76], [77].

The considerable pleiotropy (Figure 3) and epistasis (Figure 4) in the *cis*-regulatory elements of *unc-47* revealed that the same sequences responsible for misexpression of the *C. briggsae* promoter in *C. elegans* also control expression in other cells, such as AVL, RIS, and DVB (Figure 3, Figure 4, Figure S5). Their pleiotropic effects, detectable only in our experimental paradigm, were to drive different levels of expression in SDQR/L in the *C. elegans trans* background. Ectopic expression that is mediated by the same regulatory elements that control endogenous expression has been reported before [78]. Far from being experimental artifacts, differences between heterologous transgene expression and endogenous expression reveal coevolution between interacting components of the regulatory machinery [36]. Simulations show that selection on one trait can affect the genetic basis of traits that share common regulation [79]. Our results highlight the utility of reciprocal transgenics in uncovering the likely ubiquitous coevolution between components of gene regulatory systems, underlying both divergent and apparently conserved traits. Because divergence between orthologous *cis*-regulatory elements is likely to be subtle [80], [81], detailed, focused, single-gene analyses will be required to understand this process.

Our findings contribute to a growing appreciation of the importance of *cis*-*trans* coevolution [8], [75], [82]–[85]. One manifestation of coevolution is promoter restructuring [86], [87] that is evident in functional comparisons of orthologous *cis*-regulatory elements [2], [67], [80], [88], [89]. Expression of *C. elegans* and *C. briggsae* promoters in heterologous *trans* environments showed differences (Figure 1C), implying that coevolved changes underlie their conserved endogenous patterns. Those expression differences resemble transgressive segregation [27], [30], [56], [90]–[92], which is observed for a considerable fraction of genes [55], and is commonly explained by antagonistic epistasis [90].

The importance of epistatic interactions in evolution is well established [93]. Epistasis has been documented not only between unlinked loci, but also within genes. Recent experimental data indicate that complex epistatic interactions between amino acid substitutions within proteins have played an important role in shaping protein evolution [69], [70], [94], [95], particularly by constraining the order of mutations [96], [97]. Because transcription involves orchestrated interactions of different molecules, epistasis is likely to be an important force in evolution of gene regulation [87]. This view is supported by theoretical considerations [98], [99] and empirical data [2], [67], [100]–[102]. Reciprocal swaps of Region A between *C. elegans* and *C. briggsae* (Figure 4) suggest that epistasis within *cis*-regulatory elements operates even on the scale of a few nucleotides. Redundancy in *cis*-regulatory architecture (Figure 3) may play a prominent role in mediating epistatic interactions [103], [104] perhaps by providing a permissive environment in which multiple compensatory changes can take place [84], [105]. While in some instances sequence turnover may be functionally silent, experimental [106] and theoretical [107] results suggest that this process can seed regulatory elements with novel interactions and lead to the origin of new expression patterns and potentially to adaptation.

We found remarkable acceleration of sequence divergence in the *C. briggsae* promoter of *unc-47* that is concomitant with functional divergence (Figure 5 and Figure S6). Regular turnover of binding sites would be expected to lead to a clock-like evolution of regulatory sequences [108]. Instead, the pattern of accelerated sequence divergence resembles that seen in regulatory elements under strong artificial selection [109]. Whether the divergence in the *C. briggsae* promoter was adaptive, and what sort of selection pressure it might have been responding to, is not clear. Adaptive evolution in non-coding intergenic sequences may be more common than was previously thought [110].

Our results stress why functional tests are essential for meaningful comparisons between orthologous *cis*-regulatory elements. Accelerated lineage-specific evolution of regulatory sequences has been interpreted as evidence that divergent loci encode traits unique to a given species [111]–[113]. Not only did the sequence of the *C. briggsae unc-47* promoter experience accelerated lineage-specific evolution, but when we tested it in *C. elegans*, it directed intense and consistent expression in SDQR/L. This could have suggested that the pattern of *unc-47* had diverged between the two species, possibly reflecting a morphological or physiological adaptation. Analysis of reciprocal transgenics, however, showed that the expression pattern of *unc-47* has been conserved in Caenorhabditis nematodes, and the accelerated divergence of the *C. briggsae cis*-regulatory element was compensated by changes in its *trans*-regulatory environment. It is therefore possible that at least some regions of accelerated sequence evolution are sites of *cis*-*trans* coevolution that do not correspond to phenotypic divergence.

Conserved expression patterns can be maintained between two species by bursts of lineage-specific coevolution in the components of regulatory pathways. These lineage-specific changes can be revealed when they are swapped out of the context in which they evolved. We have found that the relevant context of interacting molecules, as judged by the extent of coevolution we can detect, extends from the *trans*-regulatory milieu of a cell down to neighboring base pairs of DNA. Sequence change and functional change are no doubt related, but one should not be inferred on the basis of the other alone. Widespread conservation of gene expression patterns may conceal many instances of gene regulatory evolution.

## Materials and Methods

### Transgenes and strains

To generate reporter transgenes, promoter sequences were PCR amplified from genomic DNA and cloned upstream of GFP into the Fire lab vector pPD95.75. In all cases, the start codon of the *unc-47* ortholog was included in the fusion. Prior to injection, all transgenes were sequenced to ensure accuracy. We injected a mixture (5 ng/µL promoter::GFP plasmid, 5 ng/µL *pha-1* rescue transgene, 100 ng/µL salmon sperm DNA) into temperature-sensitive *C. elegans pha-1* (e2123) strain [114]. Transformants were selected at 25°C. The *C. briggsae* strains carrying extrachromosomal arrays were produced by injecting a mixture (5 ng/µL promoter::GFP plasmid, 5 ng/µL *Cbr-unc-119* rescue plasmid and 100 ng/µL salmon sperm DNA) into YR91 *Cbr*-*unc-119* (nm67) strain. To examine the function of transcription factor *ahr-1* we used *ahr-1* (ia03), a loss-of-function allele [62]. Extrachromosomal arrays were integrated by UV integration [115]. The *C. briggsae unc-47* promoter fusion was integrated into the YR91 strain of *C. briggsae* through bombardment [115]. MosSCI single copy integrated strains were generated following an established protocol [50].

### Microscopy

Mixed-stage populations of *C. elegans* carrying transgenes were grown with abundant food and L4-stage worms were selected. These were immobilized on agar slides with 10 mM sodium azide in M9 buffer. The slides were examined on a Leica DM5000B compound microscope under 400-fold magnification. Presence/absence of GFP expression in a cell was recorded only if the cell was clearly visible, unobstructed by the intestine. Worms without any visible GFP expression were assumed to have lost the transgene. Fluorescence measurements were carried out as previously described [22]. Each photograph showing worms in figures is composed of several images of the same individual capturing anterior, middle, and posterior sections.

### Site-directed mutagenesis

Two types of mutagenized promoters were generated using the QuickChangeII kit (Stratagene). To test the role of the AHR-1 core consensus motif in regulating expression in SDQR/L, we introduced two point mutations in the conserved AHR-1 consensus motif [59], changing the sequence from AACCACGCTATT to AAC**A** AC**T**CTATT (putative binding site underlined). To test the roles of the nonconserved regions upstream of the conserved motif, we swapped these sequences between the *C. elegans* and *C. briggsae unc-47* promoters via a two-step site-directed mutagenesis.

## Supporting Information

Figure S1 Consistency of SDQR/L expression between independent strains carrying extrachromosomal arrays. The distribution of expression intensity in SDQR and SDQL relative to D-type neurons is plotted. The fraction of individuals showing expression over individuals scored is indicated underneath. Individuals were only scored if their cell was clearly visible, unobstructed by the intestine. Two independent strains carrying extrachromosomal arrays were measured for (A) *C. elegans* promoter in *C. elegans*, (B) *C. briggsae* promoter in *C. elegans*, (C) *C. elegans* promoter in *C. briggsae*, (D) *C. briggsae* promoter in *C. briggsae*.
(PDF)

Figure S2 Expression driven by integrated transgenes is consistent with expression driven by extrachromosomal arrays and between independent strains. (A) For each combination of promoter and *trans*-regulatory environment, expression in SDQR and SDQL is presented. *C. elegans* is represented by straight lines, *C. briggsae* by wavy lines. Frequency of expression is represented by the width, and intensity of expression relative to D-type neurons by the height of black boxes. Compare with Figure 1C. Number of individuals expressing and total number of individuals scored is indicated underneath. Individuals were only scored if their cell was clearly visible, unobstructed by the intestine. The distribution of expression intensity in SDQR and SDQL relative to D-type neurons is plotted. The fraction of individuals showing expression over individuals scored is indicated underneath. Two independent strains carrying integrated transgenes were measured for (B) *C. elegans* promoter in *C. elegans*, (C) *C. briggsae* promoter in *C. elegans*, (D) *C. elegans* promoter in *C. briggsae*, (E) *C. briggsae* promoter in *C. briggsae*.
(PDF)

Figure S3 Expression driven by MosSCI single-copy integrated transgenes is consistent with expression driven by extrachromosomal arrays and between independent strains. (A) Expression in SDQR and SDQL is presented for both *C. elegans* and *C. briggsae* promoters in *C. elegans*. Frequency of expression is represented by the width, and intensity of expression relative to D-type neurons by the height of black boxes. Compare with Figure 1C and Figure S2A. Number of individuals expressing and total number of individuals scored is indicated underneath. Individuals were only scored if their cell was clearly visible, unobstructed by the intestine. The distribution of expression intensity in SDQR and SDQL relative to D-type neurons is plotted. The fraction of individuals showing expression over individuals scored is indicated underneath. Two independent strains carrying integrated transgenes were measured for (B) *C. elegans* promoter in *C. elegans* and (C) *C. briggsae* promoter in *C. elegans*.
(PDF)

Figure S4 *C. elegans* and *C. briggsae* promoters have a single AHR-1 core consensus motif. Sequences of the *C. elegans* and *C. briggsae* promoters of *unc-47*. A single, conserved AHR-1 core consensus motif (highlighted in red) is present in both promoters.
(PDF)

Figure S5 Expression patterns driven by chimeric *unc-47* promoters. (A) An alignment of the sequences flanking the conserved AHR-1 consensus motif (boxed in red). Regions conserved between all four species are shaded in gray. In (B–J) bold face letters represent *C. briggsae* sequence, regular font represents *C. elegans*. All transgenes were tested in *C. elegans*. Full-length *C. elegans* promoter (B) drives inconsistent expression in SDQR, no expression in SDQL, and consistent expression in DVB. Full-length *C. briggsae* promoter (C) drives consistent expression in SDQR, SDQL and DVB. *C. elegans* promoter with *C. briggsae* Region A (D) or Region B (E) drives *C. briggsae*-like expression in SDQR, *C. elegans*-like expression in SDQL, while expression in DVB is unaffected. Additionally, expression in AVL and RIS is either severely reduced or abolished. Partial replacement of *C. elegans* Region B by 10 nucleotides of *C. briggsae* Region B (F) completely abolishes expression in SDQR/L and reduces the intensity of expression in DVB. This phenotype is similar to that observed with the *C. briggsae* promoter mutated in the conserved AHR-1 consensus motif (Figure 3B in the text), indicating that these nucleotides are critical for SDQR/L and DVB expression. *C. briggsae* promoter with *C. elegans* Region A (G) or Region B (H) drives *C. briggsae*-like expression in SDQR, *C. elegans*-like expression in SDQL, while expression in DVB is unaffected. These chimeric promoters also drive strong ectopic expression in several head neurons and the pharyngeal-intestinal valve. A partial replacement of *C. briggsae* Region B by 10 nucleotides of *C. elegans* Region B (I) does not affect expression in SDQR/L in the context of the full-length promoter. However, in the context of the proximal promoter (J), the percentage of individuals expressing in SDQR is reduced and expression in SDQL is completely eliminated. Compare to Figure 3D in the text.
(PDF)

Figure S6 Pattern of sequence conservation in the proximal promoter of *unc-47*. (A) VISTA plot of primary sequence conservation in the *unc-47 cis*-regulatory regions from *C. briggsae*, *C. remanei*, and *C. brenneri* aligned to *C. elegans*. Window size = 20 bp, threshold = 70%. (B) In the alignment of the proximal promoters from *C. briggsae, C. remanei, C. brenneri*, and *C. elegans*, conserved nucleotides are shaded in gray. Position -1 is the first nucleotide upstream of the translation start site. The conserved AHR-1 core consensus motif is boxed in red. (C) Insertions, deletions, and substitutions on each lineage are depicted as black boxes. The number of lineage-specific changes was counted by two methods. In the less stringent method, for *C. briggsae*, *C. remanei*, and *C. brenneri*, sites conserved between two species but divergent in the third were counted as branch-specific. The number of affected sites and events calculated in this way are reported in Figure 5E. In a more stringent analysis, only sites that were different from a nucleotide conserved with *C. elegans* and two other species were counted as species-specific. Using this method, *C. briggsae* has 14 substitutions, one insertion of 12 nucleotides, and a single deletion. *C. remanei* has 2 substitutions and one deletion of 2 nucleotides. *C. brenneri* has 6 substitutions and 3 insertions affecting a total of 6 sites. Eight sites for which the polarity of mutations could not be determined are not represented. In the region extending upstream of position -122, *C. briggsae* has 10 substitutions, 6 insertions affecting 26 nucleotides, and one deletion of 4 nucleotides. In contrast, in this region, there were no *C. remanei* or *C. brenneri* specific events.
(PDF)

Table S1 Consistency of independent strains.
(PDF)
