# Supplementary material for: Coevolution within and between Regulatory Loci Can Preserve Promoter Function Despite Evolutionary Rate Acceleration
Source: PLoS Genet. 2012 Sep 20;8(9):e1002961. doi: 10.1371/journal.pgen.1002961 (PMC3447958; doi:10.1371/journal.pgen.1002961)
Supplement: Figure S1 — Consistency of SDQR/L expression between independent strains carrying extrachromosomal arrays. The distribution of expression intensity in SDQR and SDQL relative to D-type neurons is plotted. The fraction of individuals showing expression over individuals scored is indicated underneath. Individuals were only scored if their cell was clearly visible, unobstructed by the intestine. Two independent strains carrying extrachromosomal arrays were measured for (A) C. elegans promoter in C. elegans, (B) C. briggsae promoter in C. elegans, (C) C. elegans promoter in C. briggsae, (D) C. briggsae promoter in C. briggsae. (PDF) [file pgen.1002961.s001.pdf]

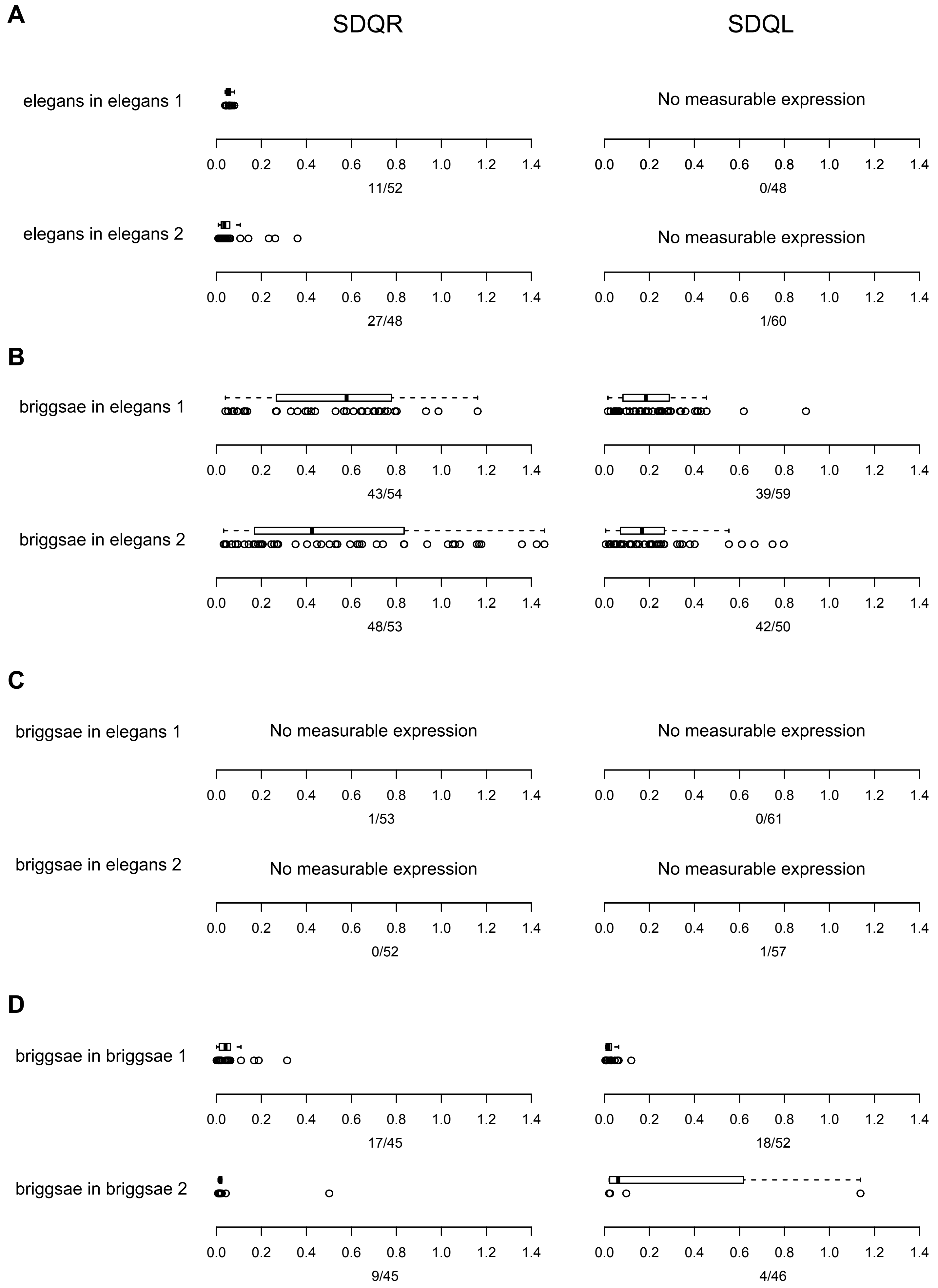

A
SDQR
SDQL
elegans in elegans 1
No measurable expression
11/52
0/48
elegans in elegans 2
No measurable expression
27/48
1/60
B
briggsae in elegans 1
43/54
39/59
briggsae in elegans 2
48/53
42/50
C
briggsae in elegans 1
No measurable expression
No measurable expression
1/53
0/61
briggsae in elegans 2
No measurable expression
No measurable expression
0/52
1/57
D
briggsae in briggsae 1
17/45
18/52
briggsae in briggsae 2
9/45
4/46

Figure S1. Consistency of SDQR/L expression between independent strains carrying extrachromosomal arrays.

The distribution of expression intensity in SDQR and SDQL relative to D-type neurons is plotted. The fraction of individuals showing expression over individuals scored is indicated underneath. Individuals were only scored if their cell was clearly visible, unobstructed by the intestine. Two independent strains carrying extrachromosomal arrays were measured for (A) *C. elegans* promoter in *C. elegans*, (B) *C. briggsae* promoter in *C. elegans*, (C) *C. elegans* promoter in *C. briggsae,* (D) *C. briggsae* promoter in *C. briggsae.*
