# Supplementary material for: Coevolution within and between Regulatory Loci Can Preserve Promoter Function Despite Evolutionary Rate Acceleration
Source: PLoS Genet. 2012 Sep 20;8(9):e1002961. doi: 10.1371/journal.pgen.1002961 (PMC3447958; doi:10.1371/journal.pgen.1002961)
Supplement: Figure S2 — Expression driven by integrated transgenes is consistent with expression driven by extrachromosomal arrays and between independent strains. (A) For each combination of promoter and trans-regulatory environment, expression in SDQR and SDQL is presented. C. elegans is represented by straight lines, C. briggsae by wavy lines. Frequency of expression is represented by the width, and intensity of expression relative to D-type neurons by the height of black boxes. Compare with Figure 1C. Number of individuals expressing and total number of individuals scored is indicated underneath. Individuals were only scored if their cell was clearly visible, unobstructed by the intestine. The distribution of expression intensity in SDQR and SDQL relative to D-type neurons is plotted. The fraction of individuals showing expression over individuals scored is indicated underneath. Two independent strains carrying integrated transgenes were measured for (B) C. elegans promoter in C. elegans, (C) C. briggsae promoter in C. elegans, (D) C. elegans promoter in C. briggsae, (E) C. briggsae promoter in C. briggsae. (PDF) [file pgen.1002961.s002.pdf]

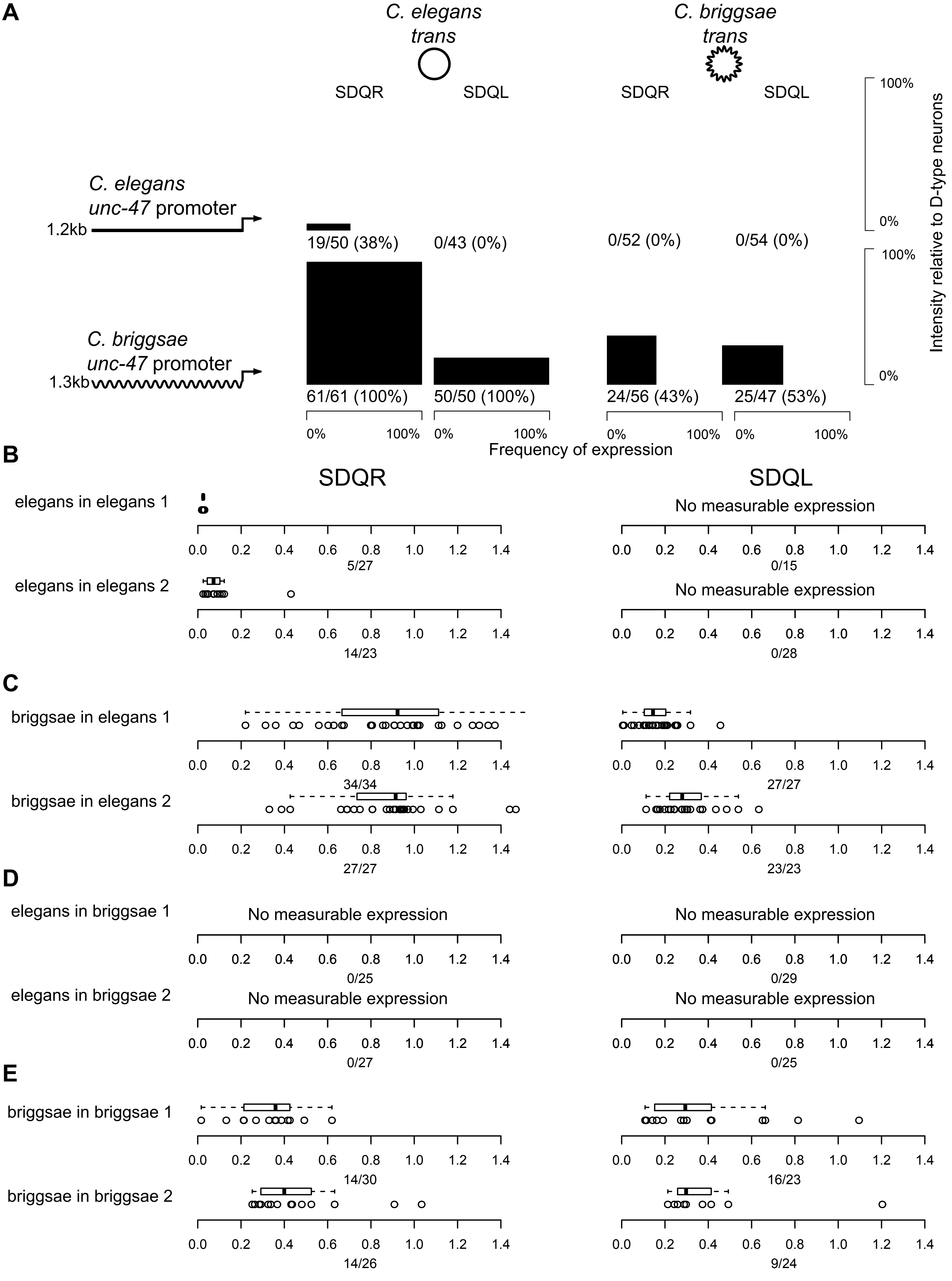
A
C. elegans trans
C. briggsae trans
SDQR
SDQL
SDQR
SDQL
100%
0%
100%
0%
Intensity relative to D-type neurons
C. elegans unc-47 promoter
1.2kb
19/50 (38%)
0/43 (0%)
0/52 (0%)
0/54 (0%)
C. briggsae unc-47 promoter
1.3kb
61/61 (100%)
50/50 (100%)
24/56 (43%)
25/47 (53%)
0%
100%
Frequency of expression
B
SDQR
SDQL
elegans in elegans 1
No measurable expression
5/27
0/15
elegans in elegans 2
No measurable expression
14/23
0/28
C
briggsae in elegans 1
34/34
27/27
briggsae in elegans 2
27/27
23/23
D
elegans in briggsae 1
No measurable expression
No measurable expression
0/25
0/29
elegans in briggsae 2
No measurable expression
No measurable expression
0/27
0/25
E
briggsae in briggsae 1
14/30
16/23
briggsae in briggsae 2
14/26
9/24
0.0 0.2 0.4 0.6 0.8 1.0 1.2 1.4

Figure S2. Expression driven by integrated transgenes is consistent with expression driven by extrachromosomal arrays and between independent strains.

(A) For each combination of promoter and *trans*-regulatory environment, expression in SDQR and SDQL is presented. *C. elegans* is represented by straight lines, *C. briggsae* by wavy lines. Frequency of expression is represented by the width, and intensity of expression relative to D-type neurons by the height of black boxes. Compare with Figure 1C. Number of individuals expressing and total number of individuals scored is indicated underneath. Individuals were only scored if their cell was clearly visible, unobstructed by the intestine. The distribution of expression intensity in SDQR and SDQL relative to D-type neurons is plotted. The fraction of individuals showing expression over individuals scored is indicated underneath. Two independent strains carrying integrated transgenes were measured for (B) *C. elegans* promoter in *C. elegans*, (C) *C. briggsae* promoter in *C. elegans*, (D) *C. elegans* promoter in *C. briggsae,* (E) *C. briggsae* promoter in *C. briggsae.*
