# Supplementary material for: Coevolution within and between Regulatory Loci Can Preserve Promoter Function Despite Evolutionary Rate Acceleration
Source: PLoS Genet. 2012 Sep 20;8(9):e1002961. doi: 10.1371/journal.pgen.1002961 (PMC3447958; doi:10.1371/journal.pgen.1002961)
Supplement: Figure S3 — Expression driven by MosSCI single-copy integrated transgenes is consistent with expression driven by extrachromosomal arrays and between independent strains. (A) Expression in SDQR and SDQL is presented for both C. elegans and C. briggsae promoters in C. elegans. Frequency of expression is represented by the width, and intensity of expression relative to D-type neurons by the height of black boxes. Compare with Figure 1C and Figure S2A. Number of individuals expressing and total number of individuals scored is indicated underneath. Individuals were only scored if their cell was clearly visible, unobstructed by the intestine. The distribution of expression intensity in SDQR and SDQL relative to D-type neurons is plotted. The fraction of individuals showing expression over individuals scored is indicated underneath. Two independent strains carrying integrated transgenes were measured for (B) C. elegans promoter in C. elegans and (C) C. briggsae promoter in C. elegans. (PDF) [file pgen.1002961.s003.pdf]

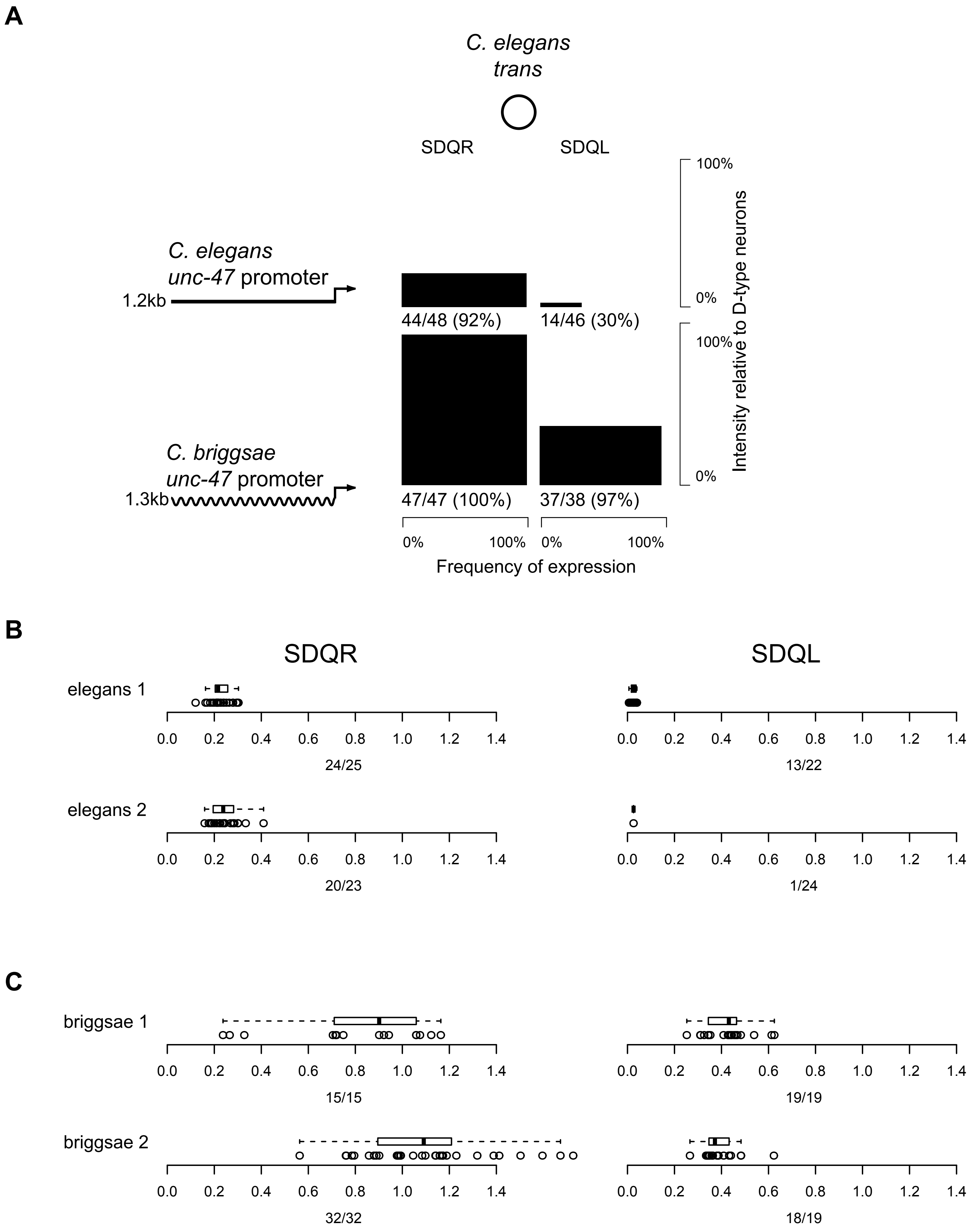
A
C. elegans
trans
SDQR
SDQL
100%
0%
100%
0%
Intensity relative to D-type neurons
C. elegans
unc-47 promoter
1.2kb
44/48 (92%)
14/46 (30%)
C. briggsae
unc-47 promoter
1.3kb
47/47 (100%)
37/38 (97%)
0%
100%
0%
100%
Frequency of expression
B
SDQR
SDQL
elegans 1
24/25
13/22
elegans 2
20/23
1/24
C
briggsae 1
15/15
19/19
briggsae 2
32/32
18/19
0.0
0.2
0.4
0.6
0.8
1.0
1.2
1.4

Figure S3. Expression driven by MosSCI single-copy integrated transgenes is consistent with expression driven by extrachromosomal arrays and between independent strains.
(A) Expression in SDQR and SDQL is presented for both *C. elegans* and *C. briggsae* promoters in *C. elegans*. Frequency of expression is represented by the width, and intensity of expression relative to D-type neurons by the height of black boxes. Compare with Figures 1C and S2A. Number of individuals expressing and total number of individuals scored is indicated underneath. Individuals were only scored if their cell was clearly visible, unobstructed by the intestine. The distribution of expression intensity in SDQR and SDQL relative to D-type neurons is plotted. The fraction of individuals showing expression over individuals scored is indicated underneath. Two independent strains carrying integrated transgenes were measured for (B) *C. elegans* promoter in *C. elegans* and (C) *C. briggsae* promoter in *C. elegans*.
