# Supplementary material for: Coevolution within and between Regulatory Loci Can Preserve Promoter Function Despite Evolutionary Rate Acceleration
Source: PLoS Genet. 2012 Sep 20;8(9):e1002961. doi: 10.1371/journal.pgen.1002961 (PMC3447958; doi:10.1371/journal.pgen.1002961)
Supplement: Figure S4 — C. elegans and C. briggsae promoters have a single AHR-1 core consensus motif. Sequences of the C. elegans and C. briggsae promoters of unc-47. A single, conserved AHR-1 core consensus motif (highlighted in red) is present in both promoters. (PDF) [file pgen.1002961.s004.pdf]

>Cel unc-47
ATCCCGGAACAGTCGAAAGTCGGTGGCAAGCGCCGAACTGCTGACGGTCTAACCGGGGCACAAATCAGGGGTGAGCGGCAAAC
GATTTTTCCGGCAAATCGGCAAATCGGCAAATTGCCAATATTGAAATACCCGGCAAATCGGTAAATAGCCGGAATTGAAAATT
TCCGGCAAACTGGTAAACCGCAAATTGCTGATTTGCCGAATTTGCCGGGAAGACGGCAATTGCCAAACATATTCGGCAAATTG
TGGTTTTGCACTTTTTGGAAATTTCAGAATTTCAATCGGCAAATTGTGCACATCCTATGAATTTTCCTACATCTATTTTGAAA
AGTAAGCAAATTCTATGAAAATATCTAAAGAAAAATGGAAAAAATTTTCAAAAAGGCACAGTTTTAAGTGTTTCCGTCTAATA
AAAAAATCCCCCTAAACACTTCCGGCAAATTGATGTTCGGCAAATGGCAAATCGGAAACTTGCCGAAAATTACAGTTTCCGGT
AAATCGGCAAACCGGCAAACTGCCTGAATTGAAAAGTTCCGTCAAATCGGCAAACCGACAACACCCCTGGCACAAATGATGGA
CATACTGAGGCAATTTGCCGGTTTTCCAATTGCAGGAAATTTTCAATTCCGGCAGTGTGCCGATTTGCCGGAAATTTTAATTC
AGGCAAATTGCCGATTTCCCGATTTCCCGATTTGCCGGAAAAAATCGTTTGCCGCCCACCCCTGGGTCTGAACCTTGATTGTT
ACAAAACATTTTTAGCTCTTTGGAGAAATAAAATGAATCTCGTAAAATTTAATTGACGAGGACGATATTAGCTGTCTCTTTAG
ACCAAATTCAGAAAAAAAGAAAGAATACTTCCCAAATTTCCGGTCCCTCTCTCGTTTTTTTTGCCAATAAACTCACTATAGTC
GCTGGTTCCCCCCTATTCACATTTATTCTACCAATCCATCAGTGGAACCAGAAAAAAGAAGAGCCTTTCGGTTTGGAGAGTAG
GGTCTAATAATCCCCCGTGCTCTTCAAATCATTGTGCCAACACACAGACACACTTTATGTGTGCTCACACACA**CACGC**TATTT
GAAGAGCGAAGACGACGACGACGCATTCAGAGCTCTTTTCCACGAAATTTGCTCCATCTTTCCACAATCTGTCTTTCCTGTGA
GACGACAGCGTCACATTTATTTCATTACAG

>Cbr unc-47
GGGATTCGGAGAACAGTAACTCAAAAGCTCGAAATATGATTCTTCAACTTTTCAAAGTTTTCTTTGATATACACAGGTAGAGG
GGCATTAAACTGCTAAATGACAGCCAAAGAGGAGCAAAATGGCGAATGACTGCTAGTTGGAAGCCGAAGGAGGACCGATAGAC
TGTCAACGGACTGTGGACGGACAACCGGAACCTCTTGGAAAACCGACTTGAAAGTTTTGATTGAAAATCATCCAGAAAAGATT
ATGTTATGCTTCCACTAGATTATTCTGATGATAGAAACTACTTTTTCTCACATAAGACAACCTCAAAAAGTATATTGTTGAAC
GACGGCTTGGTGACAGTCGAAAGGCACTGACTAGTCGATTACCGGTGGTTGGTGGACCAAATTTCTGGAGAATGGAACATTTT
GTCAAAAATTACTTTTGGAATTATCACACTATAAAAGTCAAAGAGGACTCTGGGAATCTTCAAAATTTATTATTAGAAGTATG
CGGACAAAAATGACCCATTTATATTCAGAAGAAATCTCGAAAACGTTTTTTTTTTGAAAACCAAGAGAAGAATGCGCCTGAAC
AGTGATGCTTATCGAGGTAATATAAAGACTTCAGGACTTAACAATAAAACAAATGCGTCCAGGAATTTTTGAAGCGGGTCTAG
CCAATGTTTTTTTTCTTTTTCAAAATCCCTTTCTGTGAAAAATGATGCCGTGAGATGAAGTCCCAAATTTAGAAAAGTTTCAA
ACATTGAAACAACACATTTGAGGAATAGGCTCTTCTTATTTTAGTAGCCAAGCAACAAACGAATTTCCATGCAACCCATCCGT
AGACATCAAAACGATGCTCATCTCACAGTCGTCACGTTTTTCTGAGAATGAAGAGAAACAAAAAAGGGGTTGGAAAGAAATCG
AATAGGAAATAGGGGATTACTCAATTCCAATCCCAACCGAATCTCATTTCAAAAAACTCTCAACCAACTAATCCCACATTGCC
TCGAGATTCTTTTTTCTGTAATGTGTTTCTGAATGGACTGTAAGATTGATGTTCTCATTTCCTTTTTCTCTAAAAAACCAGCC
TTCAAATTTCCGGAGCACAAGTCCCTCAACCCCAATAGGTTCGCTCCTTTCCTCTTTTCCTTATTCATTCCACTCATCCATCA
GGGAACCAAAAAGAAAACGAATAAGAAGAAGAGTCTTCGGAGAAGAGCGTCTAATAATCCCTGCTTCAAATCATTGTGCCAAC
ACAGACACACTTTATGGGCCAGAAC**CACGC**TATTTGAAGAGCAACGACGACGATGACGAGCGCCCAAGAGGTCTCCAGAGCTC
TTTTCACAAATTCTCTTCTTTCAAAACCGGTGGTTCCTTTCAAGTTTGTGTTTCCTTACAGAC

Figure S4. *C. elegans* and *C. briggsae* promoters have a single AHR-1 core consensus motif.
Sequences of the *C. elegans* and *C. briggsae* promoters of *unc-47*. A single, conserved AHR-1 core consensus motif (highlighted in red) is present in both promoters.
