# Supplementary material for: Coevolution within and between Regulatory Loci Can Preserve Promoter Function Despite Evolutionary Rate Acceleration
Source: PLoS Genet. 2012 Sep 20;8(9):e1002961. doi: 10.1371/journal.pgen.1002961 (PMC3447958; doi:10.1371/journal.pgen.1002961)
Supplement: Figure S5 — Expression patterns driven by chimeric unc-47 promoters. (A) An alignment of the sequences flanking the conserved AHR-1 consensus motif (boxed in red). Regions conserved between all four species are shaded in gray. In (B–J) bold face letters represent C. briggsae sequence, regular font represents C. elegans. All transgenes were tested in C. elegans. Full-length C. elegans promoter (B) drives inconsistent expression in SDQR, no expression in SDQL, and consistent expression in DVB. Full-length C. briggsae promoter (C) drives consistent expression in SDQR, SDQL and DVB. C. elegans promoter with C. briggsae Region A (D) or Region B (E) drives C. briggsae-like expression in SDQR, C. elegans-like expression in SDQL, while expression in DVB is unaffected. Additionally, expression in AVL and RIS is either severely reduced or abolished. Partial replacement of C. elegans Region B by 10 nucleotides of C. briggsae Region B (F) completely abolishes expression in SDQR/L and reduces the intensity of expression in DVB. This phenotype is similar to that observed with the C. briggsae promoter mutated in the conserved AHR-1 consensus motif (Figure 3B in the text), indicating that these nucleotides are critical for SDQR/L and DVB expression. C. briggsae promoter with C. elegans Region A (G) or Region B (H) drives C. briggsae-like expression in SDQR, C. elegans-like expression in SDQL, while expression in DVB is unaffected. These chimeric promoters also drive strong ectopic expression in several head neurons and the pharyngeal-intestinal valve. A partial replacement of C. briggsae Region B by 10 nucleotides of C. elegans Region B (I) does not affect expression in SDQR/L in the context of the full-length promoter. However, in the context of the proximal promoter (J), the percentage of individuals expressing in SDQR is reduced and expression in SDQL is completely eliminated. Compare to Figure 3D in the text. (PDF) [file pgen.1002961.s005.pdf]

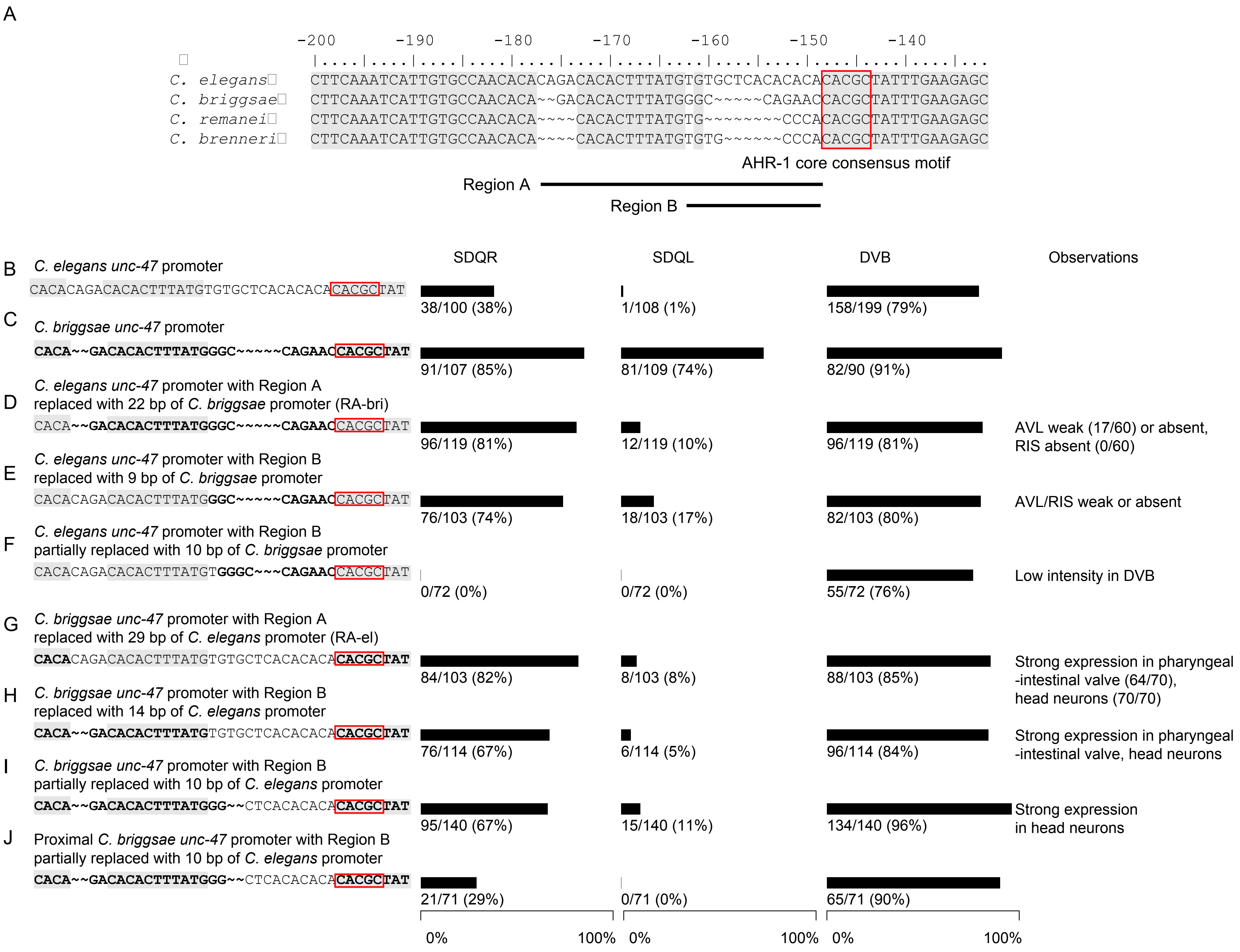
A
-200 -190 -180 -170 -160 -150 -140
C. elegans CTTCAAATCATTGTGCCAACACACAGACACACTTTATGTGTGCTCACACACACACGCTATTTGAAGAGC
C. briggsae CTTCAAATCATTGTGCCAACACA~~GACACACTTTATGGGC~~~~~CAGAACCACGCTATTTGAAGAGC
C. remanei CTTCAAATCATTGTGCCAACACA~~~~CACACTTTATGTG~~~~~~~~CCCACACGCTATTTGAAGAGC
C. brenneri CTTCAAATCATTGTGCCAACACA~~~~CACACTTTATGTGTG~~~~~~CCCACACGCTATTTGAAGAGC
AHR-1 core consensus motif
Region A
Region B
SDQR
SDQL
DVB
Observations
B
C. elegans unc-47 promoter
CACACAGACACACTTTATGTGTGCTCACACACACACGCTAT
38/100 (38%)
1/108 (1%)
158/199 (79%)
C
C. briggsae unc-47 promoter
CACA~~GACACACTTTATGGGC~~~~~CAGAACCACGCTAT
91/107 (85%)
81/109 (74%)
82/90 (91%)
D
C. elegans unc-47 promoter with Region A replaced with 22 bp of C. briggsae promoter (RA-bri)
CACA~~GACACACTTTATGGGC~~~~~CAGAACCACGCTAT
96/119 (81%)
12/119 (10%)
96/119 (81%)
AVL weak (17/60) or absent, RIS absent (0/60)
E
C. elegans unc-47 promoter with Region B replaced with 9 bp of C. briggsae promoter
CACACAGACACACTTTATGGGC~~~~~CAGAACCACGCTAT
76/103 (74%)
18/103 (17%)
82/103 (80%)
AVL/RIS weak or absent
F
C. elegans unc-47 promoter with Region B partially replaced with 10 bp of C. briggsae promoter
CACACAGACACACTTTATGTGGGC~~~CAGAACCACGCTAT
0/72 (0%)
0/72 (0%)
55/72 (76%)
Low intensity in DVB
G
C. briggsae unc-47 promoter with Region A replaced with 29 bp of C. elegans promoter (RA-el)
CACACAGACACACTTTATGTGTGCTCACACACACACGCTAT
84/103 (82%)
8/103 (8%)
88/103 (85%)
Strong expression in pharyngeal -intestinal valve (64/70), head neurons (70/70)
H
C. briggsae unc-47 promoter with Region B replaced with 14 bp of C. elegans promoter
CACA~~GACACACTTTATGTGTGCTCACACACACACGCTAT
76/114 (67%)
6/114 (5%)
96/114 (84%)
Strong expression in pharyngeal -intestinal valve, head neurons
I
C. briggsae unc-47 promoter with Region B partially replaced with 10 bp of C. elegans promoter
CACA~~GACACACTTTATGGG~~CTCACACACACACGCTAT
95/140 (67%)
15/140 (11%)
134/140 (96%)
Strong expression in head neurons
J
Proximal C. briggsae unc-47 promoter with Region B partially replaced with 10 bp of C. elegans promoter
CACA~~GACACACTTTATGGG~~CTCACACACACACGCTAT
21/71 (29%)
0/71 (0%)
65/71 (90%)
0% 100% 0% 100% 0% 100%

Figure S5. Expression patterns driven by chimeric *unc-47* promoters.

(A) An alignment of the sequences flanking the conserved AHR-1 consensus motif (boxed in red). Regions conserved between all four species are shaded in gray. In (B-J) bold face letters represent *C. briggsae* sequence, regular font represents *C. elegans*. All transgenes were tested in *C. elegans*. Full-length *C. elegans* promoter (B) drives inconsistent expression in SDQR, no expression in SDQL, and consistent expression in DVB. Full-length *C. briggsae* promoter (C) drives consistent expression in SDQR, SDQL and DVB. *C. elegans* promoter with *C. briggsae* Region A (D) or Region B (E) drives *C. briggsae*-like expression in SDQR, *C. elegans*-like expression in SDQL, while expression in DVB is unaffected. Additionally, expression in AVL and RIS is either severely reduced or abolished. Partial replacement of *C. elegans* Region B by 10 nucleotides of *C. briggsae* Region B (F) completely abolishes expression in SDQR/L and reduces the intensity of expression in DVB. This phenotype is similar to that observed with the *C. briggsae* promoter mutated in the conserved AHR-1 consensus motif (Figure 3B in the text), indicating that these nucleotides are critical for SDQR/L and DVB expression. *C. briggsae* promoter with *C. elegans* Region A (G) or Region B (H) drives *C. briggsae*-like expression in SDQR, *C. elegans*-like expression in SDQL, while expression in DVB is unaffected. These chimeric promoters also drive strong ectopic expression in several head neurons and the pharyngeal-intestinal valve. A partial replacement of *C. briggsae* Region B by 10 nucleotides of *C. elegans* Region B (I) does not affect expression in SDQR/L in the context of the full-length promoter. However, in the context of the proximal promoter (J), the percentage of individuals expressing in SDQR is reduced and expression in SDQL is completely eliminated. Compare to Figure 3D in the text.
