# Supplementary material for: Coevolution within and between Regulatory Loci Can Preserve Promoter Function Despite Evolutionary Rate Acceleration
Source: PLoS Genet. 2012 Sep 20;8(9):e1002961. doi: 10.1371/journal.pgen.1002961 (PMC3447958; doi:10.1371/journal.pgen.1002961)
Supplement: Figure S6 — Pattern of sequence conservation in the proximal promoter of unc-47. (A) VISTA plot of primary sequence conservation in the unc-47 cis-regulatory regions from C. briggsae, C. remanei, and C. brenneri aligned to C. elegans. Window size = 20 bp, threshold = 70%. (B) In the alignment of the proximal promoters from C. briggsae, C. remanei, C. brenneri, and C. elegans, conserved nucleotides are shaded in gray. Position -1 is the first nucleotide upstream of the translation start site. The conserved AHR-1 core consensus motif is boxed in red. (C) Insertions, deletions, and substitutions on each lineage are depicted as black boxes. The number of lineage-specific changes was counted by two methods. In the less stringent method, for C. briggsae, C. remanei, and C. brenneri, sites conserved between two species but divergent in the third were counted as branch-specific. The number of affected sites and events calculated in this way are reported in Figure 5E. In a more stringent analysis, only sites that were different from a nucleotide conserved with C. elegans and two other species were counted as species-specific. Using this method, C. briggsae has 14 substitutions, one insertion of 12 nucleotides, and a single deletion. C. remanei has 2 substitutions and one deletion of 2 nucleotides. C. brenneri has 6 substitutions and 3 insertions affecting a total of 6 sites. Eight sites for which the polarity of mutations could not be determined are not represented. In the region extending upstream of position -122, C. briggsae has 10 substitutions, 6 insertions affecting 26 nucleotides, and one deletion of 4 nucleotides. In contrast, in this region, there were no C. remanei or C. brenneri specific events. (PDF) [file pgen.1002961.s006.pdf]

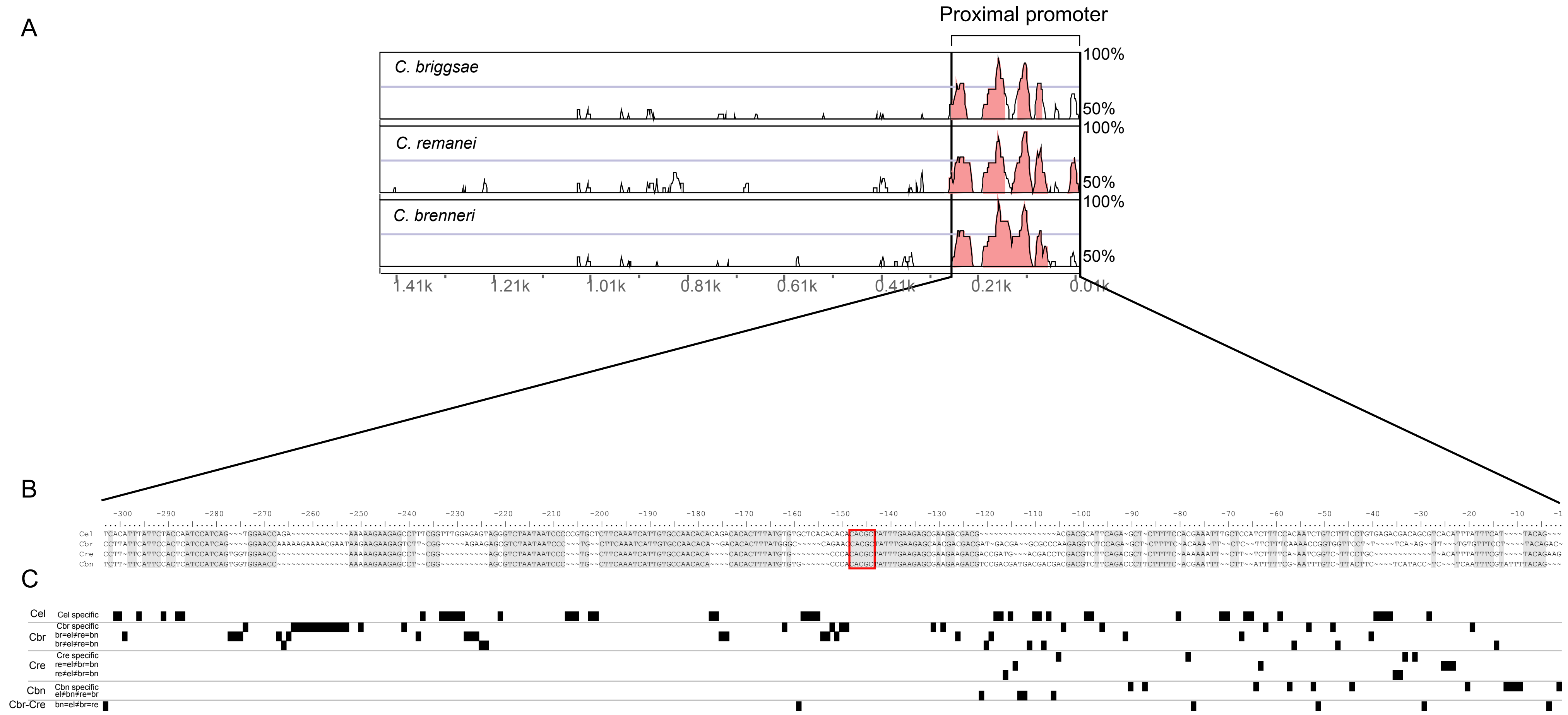

Figure S6. Pattern of sequence conservation in the proximal promoter of *unc-47*.
(A) VISTA plot of primary sequence conservation in the *unc-47 cis*-regulatory regions from *C. briggsae*, *C. remanei,* and *C. brenneri* aligned to *C. elegans*. Window size = 20 bp, threshold = 70%. (B) In the alignment of the proximal promoters from *C. briggsae, C. remanei, C. brenneri,* and *C. elegans*, conserved nucleotides are shaded in gray. Position -1 is the first nucleotide upstream of the translation start site. The conserved AHR-1 core consensus motif is boxed in red. (C) Insertions, deletions, and substitutions on each lineage are depicted as black boxes. The number of lineage-specific changes was counted by two methods. In the less stringent method, for *C. briggsae*, *C. remanei,* and *C. brenneri*, sites conserved between two species but divergent in the third were counted as branch-specific. The number of affected sites and events calculated in this way are reported in Figure 5E. In a more stringent analysis, only sites that were different from a nucleotide conserved with *C. elegans* and two other species were counted as species-specific. Using this method, *C. briggsae* has 14 substitutions, one insertion of 12 nucleotides, and a single deletion. *C. remanei* has 2 substitutions and one deletion of 2 nucleotides. *C. brenneri* has 6 substitutions and 3 insertions affecting a total of 6 sites. Eight sites for which the polarity of mutations could not be determined are not represented. In the region extending upstream of position -122, *C. briggsae* has 10 substitutions, 6 insertions affecting 26 nucleotides, and one deletion of 4 nucleotides. In contrast, in this region, there were no *C. remanei* or *C. brenneri* specific events.
