# Supplementary material for: Coevolution within and between Regulatory Loci Can Preserve Promoter Function Despite Evolutionary Rate Acceleration
Source: PLoS Genet. 2012 Sep 20;8(9):e1002961. doi: 10.1371/journal.pgen.1002961 (PMC3447958; doi:10.1371/journal.pgen.1002961)
Supplement: Table S1 — Consistency of independent strains. (PDF) [file pgen.1002961.s007.pdf]

Strains	animals expressing in SDQR/total	animals expressing in SDQL/total	animals expressing in DVB/total
**Figure 3**			
777bp strain 1	37/49	22/35	44/49
777bp strain 2	39/47	38/53	81/99
777bp mut strain 1	0/39	0/28	38/50
777bp mut strain 2	0/30	0/30	33/43
254bp strain 1	30/52	2/45	90/98
254bp strain 2	17/55	0/45	62/99
254bp mut strain1	0/71	0/71	3/71
254bp mut strain 2	0/69	0/69	2/69
**Figure 4**			
RA-el strain 1	54/60	3/60	
RA-el strain 2	30/43	5/43	
RA-bri strain 1	35/44	5/44	
RA-bri strain 2	61/75	7/75	
**Figure 5**			
Cbre full length strain 1	6/100	6/100	
Cbre full length strain 2	3/100	13/100	
Cbre proximal strain 1	0/100	0/100	
Cbre proximal strain 2	1/100	1/100	
Cre full length strain 1	2/100	0/100	
Cre full length strain 2	1/100	1/100	
Cre proximal strain 1	1/100	0/100	
Cre proximal strain 2	3/100	0/100	

Table S1. Consistency of independent strains.
